# Advanced Extracellular Vesicle Isolation: A Hybrid Electrokinetic-Tangential Flow Filtration Approach for Improved Yield, Purity, and Scalability

**DOI:** 10.1021/acs.analchem.5c01168

**Published:** 2025-07-30

**Authors:** YongWoo Kim, SoYoung Jeon, KangMin Lee, Sehyun Shin

**Affiliations:** † School of Mechanical Engineering, 34973 Korea University, Seoul 02841, Republic of Korea; ‡ Department of Micro-Nanosystem Technology, Korea University, Seoul 02841, Republic of Korea; § Engineering Research Center for Biofluid Biopsy, Seoul 02841, Republic of Korea

## Abstract

As extracellular vesicles (EVs) become increasingly important in diagnostics and therapeutics, achieving both improved purity and yield during isolation remains a critical challenge. Conventional techniques often suffer from the coisolation of nonvesicular particles and soluble proteins, limiting their clinical and research utility. In response, we introduce ExoTFF, a hybrid isolation technology that sequentially integrates electrokinetic filtration (ExoFilter) with size-exclusion tangential flow filtration (TFF) to deliver unprecedented performance gains through an iterative, synergistic mechanism. In the ExoTFF system, the sample is repeatedly circulated through an electrokinetic mesh filter and TFF until the liquid is removed. This recirculating flow gradually eliminates contaminants, while the electrokinetic filter continuously captures EVs as the sample is purified. Finally, any residual impurities in the TFF unit are completely removed via a dead volume elimination process. The complementary actions of these two distinct separation mechanisms double EV recovery rates and reduce impurity levels by 80% compared to conventional TFF, culminating in an impressive 800% improvement in the purity ratio. In proof-of-concept experiments, ExoTFF processed 10 mL of plasma within 10 min, efficiently depleting albumin and high-density lipoprotein (HDL) while achieving superior EV recovery. To further explore scalability, an automated ExoTFF system processed 500 mL of sample in 50 min, maintaining consistent yield and purity. The ability to sustain performance across different scales highlights ExoTFF’s potential for both laboratory research and industrial-level EV production. Beyond biological applications, this platform also offers broad applicability for the isolation of negatively charged nanoparticles, demonstrating its potential impact across multiple nanotechnology-driven fields.

## Graphical abstract

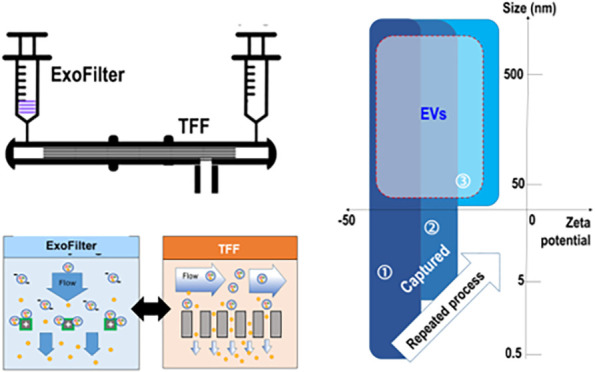

## Introduction

Extracellular vesicles (EVs), the nanoscale particles enveloped by a lipid bilayer, are secreted by all types of cells and have emerged as vital mediators of intercellular communication.^1,2^ Their ability to traverse biological barriers facilitates their roles in a variety of physiological and pathological processes, such as immune regulation, angiogenesis, and cancer metastasis.^3,4^ Although EVs are small, they possess intrinsic properties that allow them to carry an array of biomolecules—including cytoplasmic and membrane proteins, lipids, and RNA molecules—which gives them tremendous potential for various applications, including targeted therapeutic delivery.^5^ Particularly, their natural capability to convey bioactive molecules position EVs as promising cargos for delivering drugs specifically to diseased cells.^3,6^

As EVs gain increasing attention in industrial and biotechnological applications, the need for high-purity purification technologies, along with large-scale isolation techniques, has become more crucial than ever.^7^ Among current purification methods, the combination of density gradient-based ultracentrifugation (UC) and size-exclusion chromatography (SEC) is the most commonly used approach for achieving high purity in EV isolation.^8–10^ However, these technologies are designed for small-scale laboratory samples and therefore exhibit significant limitations when scaled up for industrial applications.

While ultrafiltration and Tangential Flow Filtration (TFF) are among the technologies adopted for large-scale sample extraction in industry,^11^ these methods often fall short of the purity levels required for advanced biomanufacturing.^12,13^ Since each technique has its own advantages and limitations, and operates based on different isolation principles, a trend has emerged in recent publications to combine methods such as SEC with ultrafiltration (UF).^14,15^ However, since both SEC and UF rely on the same size-based separation mechanism, these combined technologies are limited in their ability to remove impurities of similar sizes, inevitably restricting improvements in purity. In fact, nanoparticles such as high-density lipoprotein (HDL), low-density lipoprotein (LDL), and very low-density lipoprotein (VLDL), which are all abundant in plasma, have been reported to persist in EV isolates. Among them, LDL and VLDL are particularly difficult to remove due to their close similarity to EVs in both size and surface charge.^14,15^ Considering this, it is clear that a single separation technique alone cannot achieve sufficient purification. Moreover, when designing a combination of two or more techniques for purification, it is advisible to incorporate different isolation mechanisms that complement each other’s weaknesses while enhancing their strengths.^14^

Hence, this study aims to develop an innovative EV isolation technology that achieves high-level purification while maintaining high throughput. To this end, we carefully selected two technologies with different isolation mechanisms from among various available methods and integrated them through an advanced unified design into a single hybrid system, ExoTFF. The ExoTFF system consists of ExoFilter^16^ employing an electrokinetic-based separation mechanism and TFF utilizing a size exclusion principle.

To integrate these principle, this study developed an innovative syringe-type ExoFilter and connected it with a conventional cylindrical TFF system, thereby realizing a disposable ExoTFF system that integrates both technologies into one. To confirm the performance of the ExoTFF and its advantages over existing methods, we analyzed isolated EV on blood plasma samples according to ‘MISEV2023’,^17^ and compared the performance of ExoTFF with that of a single TFF or ExoFilter. We then extended the ExoTFF application to various biofluids, including urine, saliva, and culture medium. Additionally, as proof of concept for large-volume sample processing, an automated ExoTFF system was developed and demonstrated for handling 500 mL of plant extracted liquid within 10 min.

## Materials and Methods

### Design and Operating Principle of ExoTFF

To enhance the purity of extracellular vesicles (EVs), we developed ExoTFF, a hybrid platform that integrates charge-based filtration (ExoFilter) with size-based filtration (tangential flow filtration, TFF) in a single, syringe-driven device (Figure 1(a)). The two modules are interconnected via a three-way valve, allowing manual control of flow direction. This configuration enables reciprocating (bidirectional) flow, where the sample or elution buffer can circulate repeatedly between the ExoFilter and TFF modules without the need for external pumps (Figure 1(b)).

**Figure 1:**
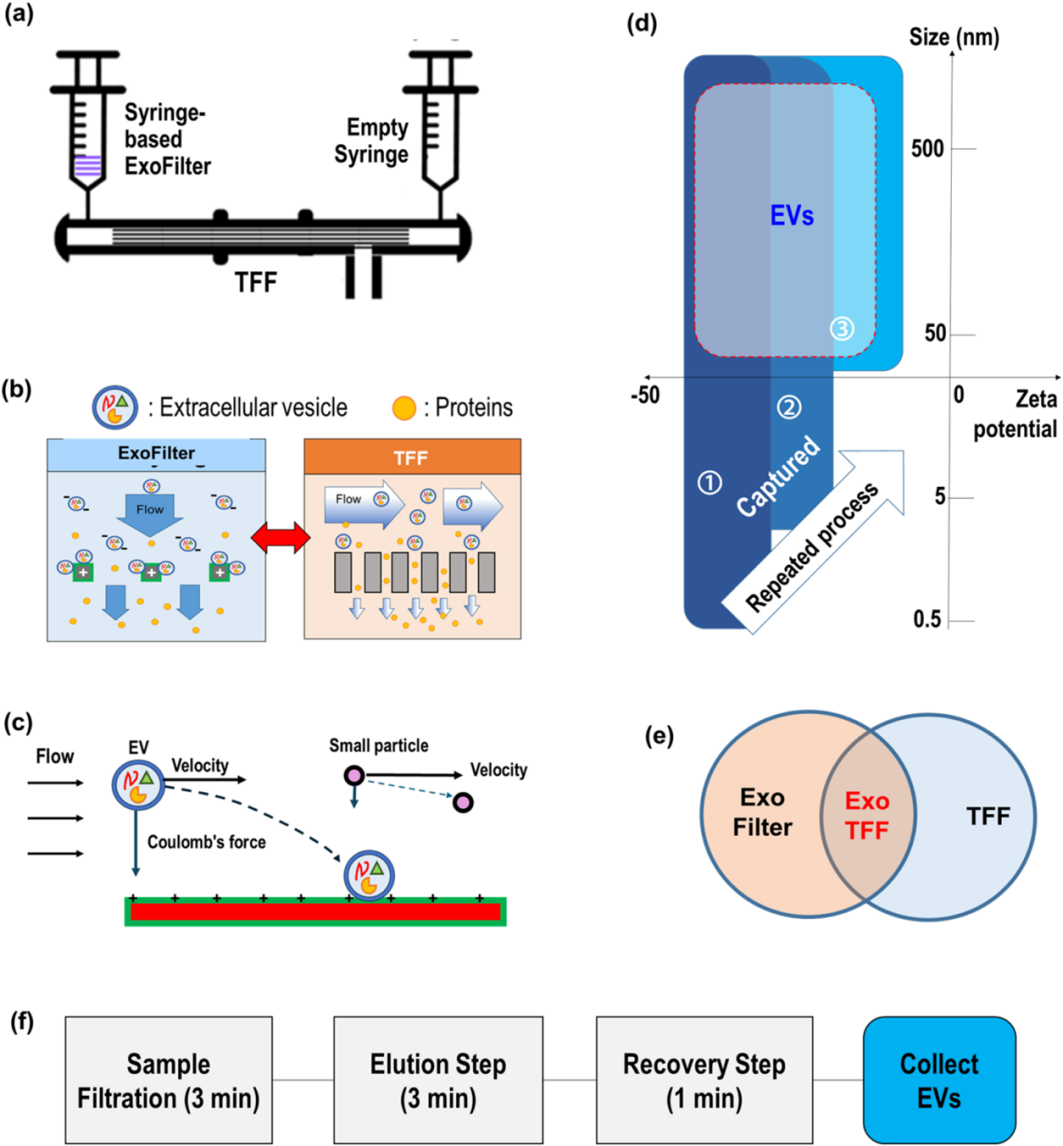
Hybridization of electrokinetic mesh filtration (ExoFilter) and size-based Tangential Flow Filtration (TFF). (a) Schematic of electrokinetic assisted mesh flow filtration with TFF, (b) dual mechanisms of ExoTFF consisting electrokinetic filtration and size exclusion filtration, (c) electrokinetic mechanism of ExoFilter for the selective capture of EVs over smaller contaminant particles, (d) operation window of ExoTFF in ζ-potential vs size domain, (e) Venn diagram of the hybrid ExoTFF system, which combines key features of ExoFilter and TFF, (f) protocol of ExoTFF consisting of sample filtration, elution and recovery processes.

The ExoFilter module consists of a positively charged multilayer mesh that selectively captures negatively charged EVs via electrokinetic interactions under flow conditions (Figure 1(c)). Unlike conventional size-based filters, the ExoFilter uses a mesh with relatively large pores (10 μm), minimizing shear stress and preserving the structural integrity of EVs. Captured EVs are gently eluted by introducing a high-salt buffer (e.g., 1 M NaCl), which disrupts electrostatic interactions between the EV membranes and the positively charged mesh. The resulting eluate containing EVs, dissolved salts, and other low-molecular-weight contaminants is then processed through a TFF module equipped with ∼50 nm pores (MWCO: 800 kDa). During this step, the high-salt elution buffer and small solutes are efficiently removed through the membrane, while the EVs are retained in the retentate. Unlike dead-end filtration, TFF’s tangential flow reduces membrane fouling and supports scalability.

During operation, the sample is first loaded into the syringe and pushed through the ExoFilter and into the TFF membrane. Flow is then reversed and pulled back into the syringe, and this cycle is repeated 4–5 times. The reciprocating motion enhances capture efficiency by increasing contact frequency between EVs and the ExoFilter surface, while simultaneously allowing TFF to continuously remove contaminants (Figure 1(d)).

Once filtration is complete, the elution buffer is introduced to release EVs from the ExoFilter. The vesicles are then retained and concentrated in the TFF module and recovered in a physiological buffer such as PBS or saline (Figure 1(g), recovery step). A key advantage of ExoTFF is that it enables simultaneous buffer exchange and volume concentration, allowing for easy adjustment for downstream applications.

By leveraging both surface charge and size selection, this dual-mode isolation strategy achieves a level of purity and structural preservation that surpasses either method alone as shown in Figure 1(f). Notably, sequencing the ExoFilter prior to TFF was found to significantly reduce protein contamination (by ∼86%) while maintaining high EV recovery (Figure S1). Together, the integrated design, bidirectional flow, and modular control of ExoTFF provide a compact, pump-free solution for high-purity EV isolation across research and clinical applications.

### Preparation of Syringe-Type ExoFilter

Nylon meshes (11 mm diameter; Lixin Huarun MESH, China) were activated via glutaraldehyde cross-linking and functionalized with protamine sulfate to impart a positive surface charge. These treated meshes were layered and fixed within a 10 mL syringe using a frit to construct a syringe-type ExoFilter. The device was connected to a three-way valve, allowing manual control of flow direction and enabling bidirectional circulation without external pumps. The syringe volume was selected to match the minimum input requirement for downstream TFF processing (≥5 mL).

### Sample Preparation

Human plasma was obtained from Zen-Bio Inc. (NC), and umbilical cord mesenchymal stem cell culture media (CCM), urine, and saliva samples were freshly collected. All samples were centrifuged at 3000*g* for 15 min and filtered through an 800 nm mesh to remove large debris and aggregates. Supernatants were stored at −80 °C until use.

### EV Isolation by ExoFilter

Samples were passed through ExoFilter under gravity (1 atm) or vacuum (−68 kPa), depending on volume. EVs were captured on the cationic mesh via electrostatic interactions and then eluted with 1 M NaCl (volume: 1/5 of input). For small volumes (1–15 mL), manual pressure and brief centrifugation (5000*g*, 1 min) were used to aid filtration.

### EV Isolation by Tangential Flow Filtration (TFF)

For size-based separation, we used a commercial hollow fiber TFF module (TFF-EVs, Hansa BioMed, Estonia; MWCO 800 kDa, ∼50 nm pore size). A 10 mL sample was loaded and passed through the system repeatedly using a syringe. After initial filtration, the module was washed with 10 mL PBS, followed by EV elution with 2 mL PBS.

### EV Isolation by ExoTFF

The ExoTFF system integrates ExoFilter and TFF in a closed-loop, syringe-operated circuit. A 10 mL sample was reciprocally flowed between the ExoFilter and TFF modules using manual piston motion. This enhanced EV capture via repeated contact and allowed continuous removal of small impurities. Elution was performed using 1 M NaCl (10 mL), and purified EVs were recovered and concentrated in 2 mL PBS (5× concentration).

### EV Isolation by UC and ExoQuick

Ultracentrifugation (UC) was conducted at 120,000*g* following standard differential centrifugation. ExoQuick (System Biosciences) and ExoPAS (Microgentas, Korea) were used for PEG-based EV precipitation. ExoPAS employed sequential treatment with protamine sulfate and PEG8000, followed by low-speed centrifugation.

### EV Isolation by SEC

EVs were isolated using a 70 nm qEV10 size-exclusion chromatography column (IZON Science, Cambridge, MA) and an automatic fraction collector-V2 (IZON Science, Cambridge, MA). Initially, the column was equilibrated at room temperature for 30 min and subsequently washed with phosphate-buffered saline (PBS). A 10 mL biofluid sample was then loaded onto the column. Seven consecutive 2 mL fractions were eluted by the addition of PBS. In accordance with the manufacturer’s protocol, the fourth fraction, which was enriched in EVs, was utilized for further analysis.

### Characterization of EVs

- SEM and TEM: EV morphology was visualized using scanning electron microscopy (Quanta 250 FEG) and transmission electron microscopy (JEM-1400 Flash) after fixation, staining, and dehydration.
- NTA: Particle size and concentration were analyzed using NanoSight NS300 (Malvern Panalytical).
- Protein Quantification: Total protein was measured via BCA assay (Pierce kit, Thermo Scientific) using BSA standards.
- Western Blot: EV markers (CD9, CD81, TSG101, ALIX), ApoA1, ApoB100, and albumin were probed via SDS-PAGE and chemiluminescence detection.
- ELISA: Sandwich ELISA targeting CD9, CD81, ApoA1and ApoB100 was performed for semiquantitative analysis for EVs and lipoproteins.
- RT-qPCR: EV-associated miRNAs (let-7a-5p, miR-142–3p) were quantified using TaqMan assays after RNA extraction.

Further methodological details are provided in the Supporting Information.

### Functional Assessments

- Cellular Uptake: PKH67-labeled EVs were incubated with human dermal fibroblasts (HDFs) and imaged with fluorescence microscopy.
- Cytotoxicity: HDF viability after EV exposure (2 × 10^9^ particles/mL for 72 h) was assessed using WST-1 assay.
- ζ-Potential: Surface charge of EVs and plasma proteins was analyzed using Zetasizer Pro and SurPASS 3 analyzers.

Further methodological details are provided in the Supporting Information.

## Results and Discussion

### Verification of EV Identity in Particles Isolated by ExoTFF

As shown in Figure 2(a–b), EVs isolated using the ExoTFF method were visualized via scanning electron microscopy (SEM) and transmission electron microscopy (TEM). The images revealed that the EVs exhibited a spherical morphology with an average diameter of approximately 155 nm and a well-defined phospholipid bilayer. Nanoparticle tracking analysis (NTA) further characterized the size distribution of the isolated EVs, ranging from 50 to 450 nm, consistent with exosomes and large EVs (Figure 2(c)).

**Figure 2:**
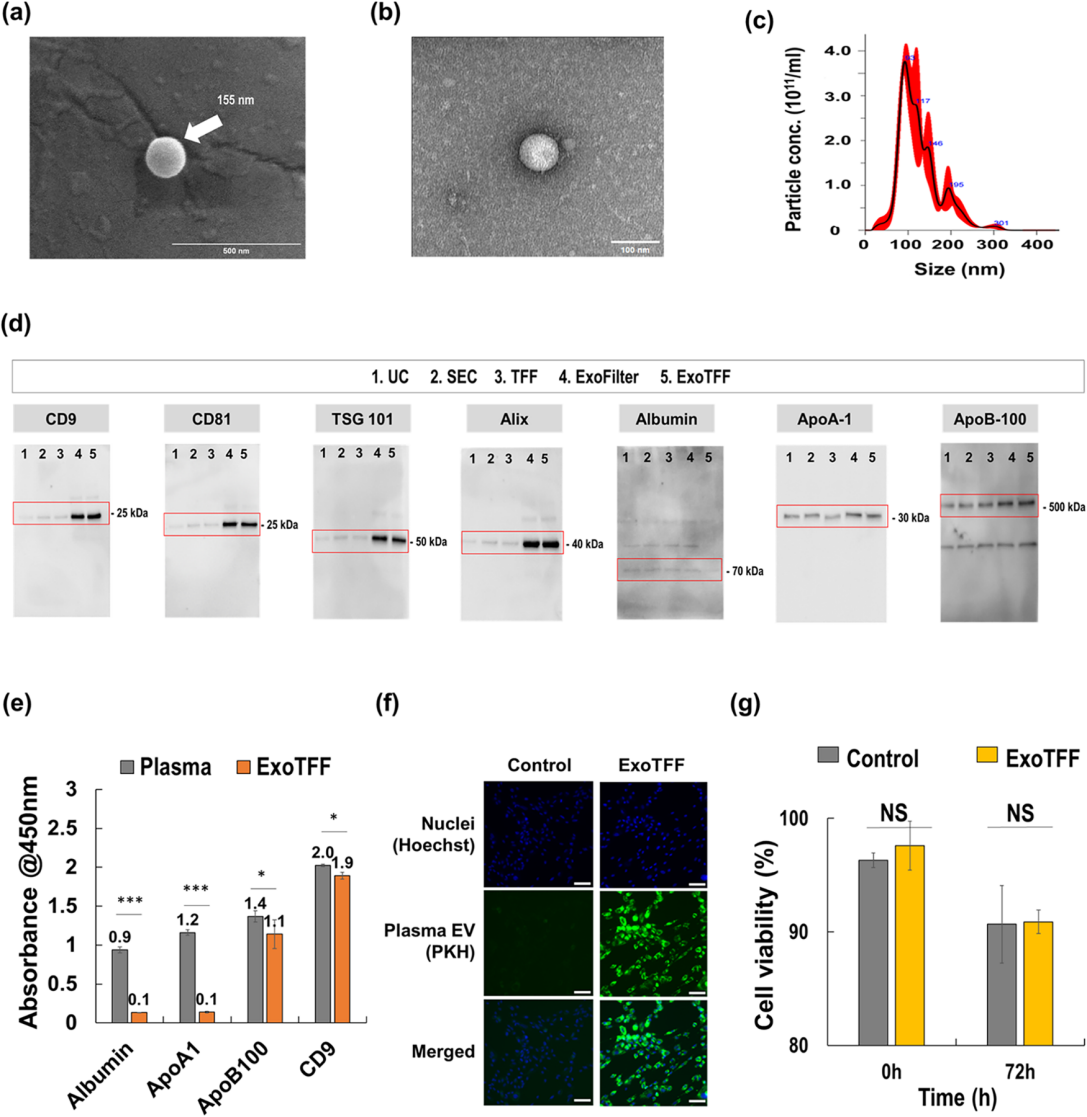
Characterization of EVs isolated from blood plasma using ExoTFF. (a) SEM image of an eluted EV, (b) TEM image of an eluted EV, (c) particle size distribution and concentrations of EVs, (d) western blot analysis of EV markers and contaminant proteins across five isolation methods (UC, SEC, TFF, ExoFIlter and ExoTFF), (e) ELISA assay results for albumin, ApoA1, ApoB100, and CD9. (*p < 0.05, ***p < 0.005), (f) cellular uptake of EVs into human dermal fibroblasts (HDFs). (g) Cytotoxicity test of EVs in HDF after 72 h.

To confirm the identity of the isolated EVs, Western blot analysis was performed to detect specific EV markers (Figure 2(d)). The presence of CD9, CD81, TSG101, and Alix—well-established EV markers—was confirmed across all methods. Notably, ExoTFF isolates exhibited strong EV marker expression alongside markedly reduced levels of contaminant proteins such as albumin, ApoA-1, and ApoB-100. Compared to conventional methods including UC, SEC, and TFF, both ExoFilter and ExoTFF showed clearer and more intense EV marker bands, indicating higher recovery efficiency of extracellular vesicles. While contaminant proteins such as albumin, ApoA-1, and ApoB-100 were present at comparable levels to those observed in UC and SEC preparations, the overall purity remained acceptable, supporting that ExoTFF achieves efficient EV isolation with enhanced yield without compromising purity.

As shown in Figure 2(e), an ELISA assay validated the presence of CD9, a representative EV surface marker, further confirming that the particles isolated by ExoTFF were indeed EVs rather than other types of nanoparticles. Based on the CD9 signal intensity, the EV recovery rate is estimated to be approximately 95%.

The assay also evaluated ApoA1, a biomarker for HDL, which is typically abundant in plasma and difficult to eliminate using many conventional isolation techniques.^23^ Notably, ApoA1 was reduced to approximately 8.3% of its original concentration following ExoTFF, despite its negative ζ-potential. In contrast, ApoB-100, a biomarker for (V)­LDL, showed a residual level of approximately 78.5%, indicating that complete removal of (V)­LDL-associated components remains challenging. These findings, in conjunction with the Western blot results in Figure 2(d), highlight a meaningful advancement, as many current EV isolation methods struggle to adequately eliminate lipoproteins and plasma proteins. This underscores the improved EV purity and recovery efficiency achieved with ExoTFF.

The functional bioactivity of EVs isolated by ExoTFF was confirmed through various assays, as depicted in Figure 2(f). Cellular uptake studies demonstrated that human dermal fibroblasts effectively internalized the EVs. Additionally, cytotoxicity assays conducted over 72 h (Figure 2(g)) showed that the EVs did not induce significant cytotoxic effects, maintaining high cell viability.^18,19^ These results confirm that the isolated EVs retain their functional bioactivity without adversely affecting the cells.

### ExoTFF Outperforms Other Methods in Yield and Purity

The superior performance of ExoTFF was demonstrated through NTA and BCA analyses of plasma-derived EVs (Figure 3(a)–(b)). Among all evaluated methods—including UC, SEC, TFF, and ExoFilter—ExoTFF achieved the highest particle concentration (5.5 × 10^11^ particles/mL), representing a 139% increase compared to TFF. In parallel, ExoTFF significantly reduced protein contamination to 6.5 mg/mL, corresponding to a 75% decrease relative to TFF. Compared to SEC, ExoTFF yielded approximately three times higher particle concentration and only half the amount of protein contaminants. It should be noted, however, that NTA measurements may include a proportion of LDL and other lipoprotein particles.^22^ Nevertheless, these results highlight that ExoTFF provides enhanced EV recovery while effectively minimizing nonvesicular protein impurities.

**Figure 3:**
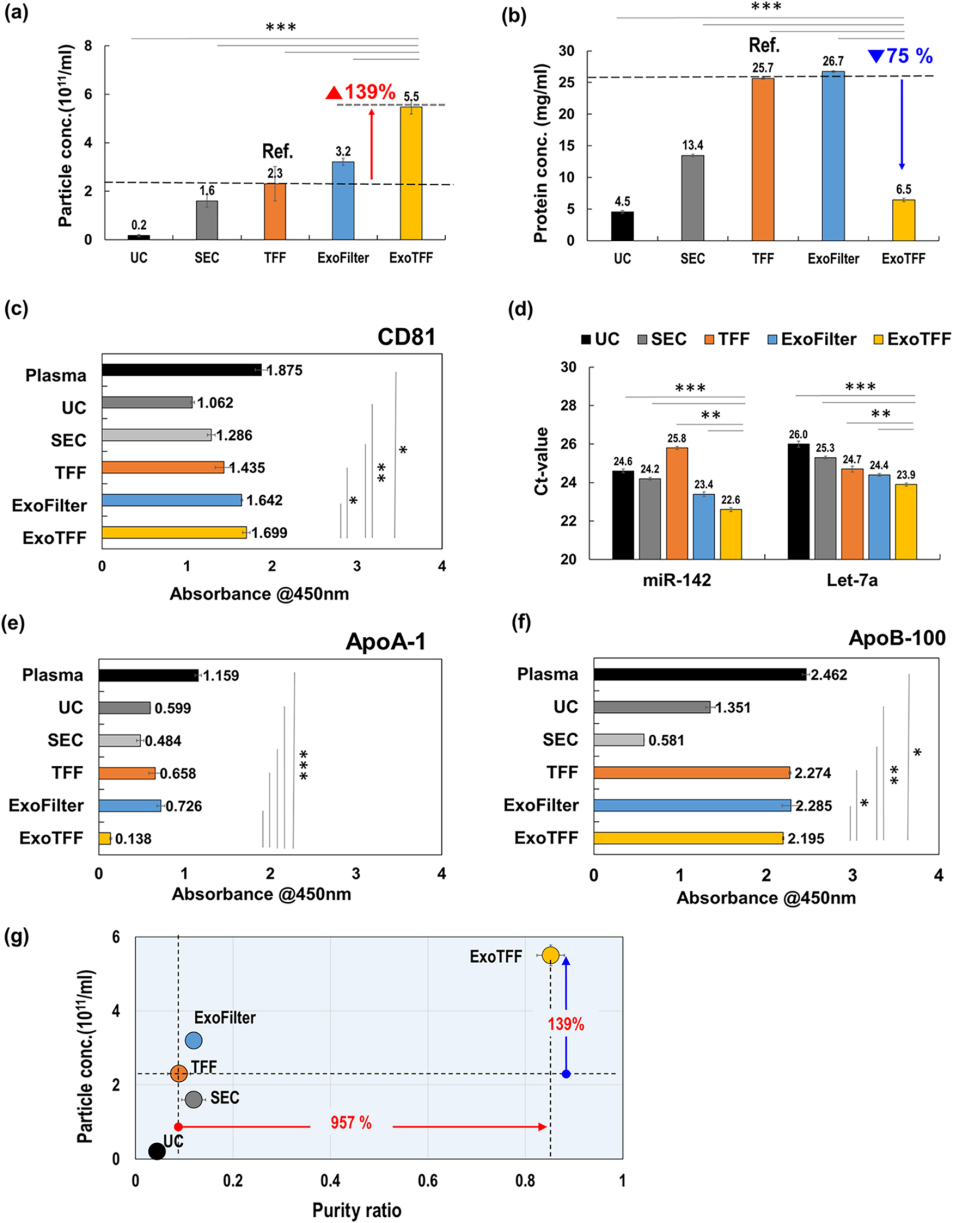
Comparative analysis of EV isolation methods (TFF, ExoFilter, ExoFilter→TFF, and ExoTFF) using plasma samples. (a) Particle concentration, (b) protein concentration of isolated EVs, (c) ELISA assays for a specific EV marker (CD81), (d) quantification of EV-derived miRNAs (miR-142 and let-7a) using RT-PCR, (e) ELISA assays for specific HDL markers (ApoA1), (f) comparison of Purity ratios for various methods. (*p < 0.05, **p < 0.01, ***p < 0.005).

The effectiveness of ExoTFF was further validated through ELISA analysis using three biomarkers: CD81 (an EV marker), ApoA1 (an HDL marker), and ApoB100 (a marker for VLDL and LDL). Plasma was used as the reference, and ExoTFF was compared with UC, SEC, TFF, and ExoFilter. Figure 3(c) shows CD81 expression levels as an indicator of EV yield. ExoTFF achieved an absorbance of 1.699, corresponding to 90.6% of the plasma reference value (1.875), indicating the highest EV recovery efficiency among the tested methods. ExoFilter and TFF also exhibited relatively high CD81 levels (87.6 and 76.5%, respectively), while UC and SEC showed lower signals (56.6 and 68.6%), suggesting comparatively reduced EV recovery.

Notably, there was a marked discrepancy between the NTA and ELISA results. NTA may overestimate EV yield due to the inclusion of similarly sized particles such as (V)­LDL, which are indistinguishable by either size or ζ-potential. In fact, according to our recent findings, as much as 60% of the particles detected by NTA were identified as lipoproteins.^22^ In contrast, ELISA detects specific surface markers through antibody binding on a fixed sensor surface, and is subject to biophysical limitations. Factors such as restricted diffusion of EVs in liquid phase, limited epitope accessibility due to steric hindrance, and size-dependent lift forces near the solid interface may reduce the probability of EVs reaching and binding to the sensor. These physical constraints can lead to underestimation or plateauing of signal intensity, thereby masking subtle differences in EV yield across isolation methods.

Therefore, in this study, we conducted a quantitative comparison of EV isolation methods by amplifying two representative miRNAs—miR-142 and let-7a—commonly used as EV housekeeping markers, using RT-qPCR. As shown in Figure 3(d), ExoTFF yielded the lowest Ct values for both miR-142 (22.6) and let-7a (23.9), indicating the highest miRNA abundance among the tested methods. In contrast, higher Ct values were observed for UC (24.6/26.0), SEC (25.3/24.2), and TFF (25.8/24.7), suggesting lower miRNA content and thus reduced EV recovery. Notably, for miR-142, the ΔCt between TFF and ExoTFF was 3.2, suggesting that ExoTFF enables more efficient recovery of EV-associated miRNAs than TFF under equal input conditions. These results highlight the superior EV enrichment efficiency of the ExoTFF process.

Figure 3(e) shows the levels of ApoA1—an established HDL biomarker—measured by ELISA to assess HDL contamination across different EV isolation methods. The plasma reference sample exhibited an absorbance of 1.159. Among the tested methods, ExoTFF showed the most effective HDL removal, reducing ApoA1 levels to 0.138, corresponding to a removal efficiency of approximately 88%. In contrast, UC and SEC yielded moderate reductions to 0.599 and 0.484, reflecting HDL removal efficiencies of approximately 48 and 58%, respectively. TFF and ExoFilter alone resulted in less efficient removal (0.658 and 0.726), corresponding to 43 and 37% removal, respectively. These results indicate that ExoTFF markedly outperforms individual methods—including SEC and TFF—in eliminating HDL-associated contaminants, offering a more selective and improved-purity EV isolation approach.

Figure 3(f) presents ELISA-based quantification of ApoB100, a representative marker for LDL and VLDL. The plasma control showed the highest absorbance at 2.462. While SEC substantially reduced ApoB100 levels to 0.581 (approximately 76% removal), all other methods—including UC, TFF, ExoFilter, and ExoTFF—showed minimal reduction, with absorbance values ranging from 1.351 to 2.285. Specifically, ExoTFF resulted in an ApoB100 level of 2.195, indicating that a large proportion of LDL/VLDL particles remained in the isolate. These results highlight the challenge of removing lipoprotein contaminants with physical properties (size and charge) similar to EVs, particularly LDL and VLDL, and underscore the need for future strategies that target lipoprotein-specific surface markers.

Figure 3(f) illustrates the relationship between particle concentration (vertical axis) and purity ratio (horizontal axis) for TFF, ExoFilter, and ExoTFF. The purity ratio is defined as the proportion of EVs relative to total protein or other contaminants, indicating how “clean” the isolated EVs are. The results show a substantial increase in both purity and yield with ExoTFF. While ExoFilter achieves a moderate improvement in particle concentration and purity ratio compared to TFF and SEC, ExoTFF demonstrates a dramatic rise: the particle concentration is 124% higher than that of TFF, and the purity ratio improves by 933%. Furthermore, ExoTFF’s hybrid process—combining selective capture and size-based filtration—yields a significantly higher purity ratio than TFF or ExoFilter alone, resulting in minimized protein contamination and maximized EV yield. Overall, Figure 3(f) underscores ExoTFF’s superior performance in delivering both high particle concentration and high purity ratio, confirming its effectiveness in producing cleaner, more concentrated EV preparations compared to TFF or ExoFilter alone.

Taken together, the results presented in Figure 3 demonstrate a clear distinction between TFF and ExoTFF in terms of EV purity and isolation efficiency. While NTA may overestimate EV particle counts due to the inclusion of similarly sized non-EV particles such as (V)­LDL, it fails to detect particles smaller than ∼20 nm, including HDL. This limitation provides critical context for interpreting the discrepancy between particle counts and protein content observed in the eluates. Specifically, although TFF samples exhibited lower particle counts (Figure 3a), they showed markedly higher total protein levels (Figure 3b) and elevated HDL concentrations as confirmed by ELISA (Figure 3e). Given that HDL particles (∼8–12 nm) are undetectable by NTA but significantly influence BCA measurements, the high protein content in TFF eluates is likely attributable to these small, protein-rich contaminants. In contrast, ExoTFF effectively removes such sub-NTA-sized impurities while enriching for EVs, resulting in both increased particle counts and an improved particle-to-protein ratio. Collectively, the NTA, BCA, and ELISA results support the superior selectivity and performance of ExoTFF in isolating high-purity EVs.

The synergistic performance of ExoTFF over conventional TFF is not simply additive, but arises from a synergistic interaction between the two modules. This synergy enables higher EV yield and improved purity, as demonstrated by increased particle counts and reduced protein contamination. To understand the synergistic mechanism behind this effect, we considered two main factors that contribute to the improved removal of protein-rich contaminants, particularly HDL, in ExoTFF.

First, ExoTFF begins with the selective electrokinetic capture of negatively charged particles, including EVs and (V)­LDL, via ExoFilter, thereby reducing matrix complexity prior to TFF. This pre-enrichment minimizes the risk of membrane fouling and competitive adsorption that are common in conventional TFF,^24^ where EVs, (V)­LDL, HDL, and various proteins coexist at high concentrations. In such fouled membranes, HDL particles (∼8–12 nm), despite their small size, are often inefficiently removed and remain trapped within the system. In contrast, ExoTFF facilitates more effective clearance of HDL and other small contaminants during the TFF step, resulting in reduced protein contamination (BCA) and enhanced EV purity.

Second, in ExoTFF, elution with a high-salt buffer (∼1 M NaCl) not only facilitates EV release from the ExoFilter but also transiently neutralizes the negative surface charge of the TFF membrane.^25^ This charge screening reduces electrostatic repulsion, thereby allowing negatively charged contaminants such as HDL to pass through more readily. This mechanism aligns with previous studies showing that negatively charged ultrafiltration membranes reject similarly charged proteins via electrostatic repulsion, an effect that can be modulated by ionic strength to improve flux and separation performance.^24^ These findings support our observation that HDL contamination is significantly reduced in ExoTFF compared to TFF alone.

### ExoTFF Outperforms across Various Biofluids

We conducted further analyses to evaluate the ability of the ExoTFF method to isolate extracellular vesicles (EVs) from various biofluids, including conditioned cell media (CCM), urine, and saliva, and compared its performance against traditional methods. For CCM (Figure 4a,b), ExoTFF demonstrated a 73% reduction in protein concentration and a 109% increase in particle concentration compared to TFF, outperforming all other EV isolation methods tested, including UC, SEC, ExoQuick, and ExoFilter.

**Figure 4:**
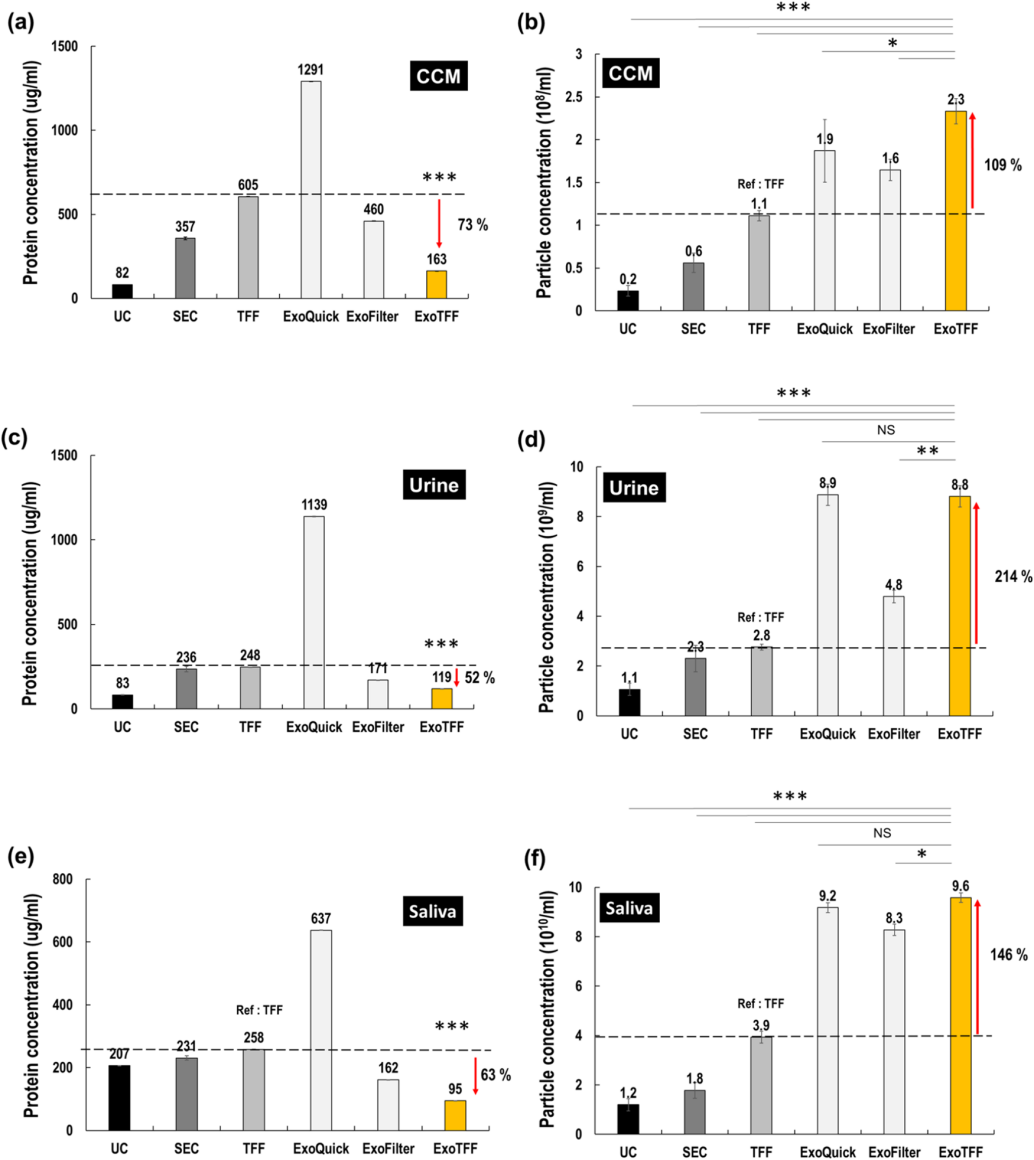
Comparison of EV isolation methods across various biofluids (CCM, urine, saliva). For protein concentration analysis (a, c, e), ExoTFF showed statistically significant reductions in contaminating protein levels compared to all other methods (***p < 0.005). For particle concentration analysis (b, d, f), statistical significance was determined between specific groups using one-way ANOVA followed by Tukey’s post hoc test (*p < 0.05, **p < 0.01, ***p < 0.005; NS = not significant).

In urine samples (Figure 4c,d), ExoTFF achieved a 52% reduction in protein concentration and a 214% increase in particle concentration compared to TFF, marking the highest performance among all methods including UC, SEC, ExoQuick, and ExoFilter. For saliva samples (Figure 4e,f), ExoTFF resulted in a 63% decrease in protein concentration and a 146% increase in particle concentration relative to TFF, similarly showing superior performance over UC, SEC, ExoQuick, and ExoFilter.

Overall, the results depicted in Figure 4 confirm that the ExoTFF method is highly effective at isolating EVs from a diverse range of biofluids, significantly enhancing both yield and purity compared to conventional isolation methods. The ExoTFF system not only demonstrates exceptional performance in blood plasma but also consistently outperforms conventional isolation methods in other biofluids, such as CCM, urine, and saliva. These findings indicate that ExoTFF is a versatile and powerful tool for EV isolation, offering broad potential utility in both research and clinical applications. Nevertheless, further experimental validation and mechanistic studies are warranted to fully elucidate the factors underlying its enhanced performance across various biofluids.

### Automated ExoTFF System for Reliable, High-Throughput EV Isolation

In this study, the ExoTFF system was implemented as an automated setup consisting of a peristaltic pump, reservoir, ExoFilter and TFF and its performance was compared to that of automated TFF and ExoFilter systems, respectively. The sample volume used for the comparison was 500 mL of CICA () plant extract. The performance comparison of the TFF, ExoFilter, and ExoTFF systems was analyzed, focusing on the operational characteristics of each system, including filtration speed, elution, recovery, and total processing time (Table 1).

**Table 1:** Operational Throughput of Three Systems Including TFF, ExoFilter and ExoTFF

CICA extract	TFF	ExoFilter	ExoTFF
sample (500 mL)	24 min	1.0 min	24 min
elution (500 mL)		0.3 min	20 min
recovery (100 mL)	1 min		1 min
total time	25 min	1.3 min	45 min
processing rate	20 mL/min	384 mL/min	11 mL/min

As a recent study reported, ExoFilter can process samples at an impressive rate of 384 mL/min using a charge-based filtration system. Additionally, the TFF system operates at a rate of 20 mL/min, focusing on size exclusion. Meanwhile, ExoTFF, which combines TFF and ExoFilter, requires a relatively longer time of 45 min to process 500 mL, resulting in a processing rate of 11 mL/min (or 660 mL/h).

The processing rate of the ExoTFF system represents a remarkable improvement compared to conventional methods. Moreover, as the ExoTFF system connects TFF and ExoFilter in series, increasing the capacity of each component can substantially enhance the system’s hourly throughput. This highlights the potential of ExoTFF to significantly improve EV purity processing in a high-throughput manner.

In this study, we introduce ExoTFF, a platform designed for high-throughput, scalable EV isolation with superior purity. Our results demonstrate high signal intensities for EV markers and miRNAs, with minimal contamination from non-EV proteins, underscoring the method’s effectiveness. By integrating two isolation principles, size and electrical charge, ExoTFF enables the selective purification of EV subpopulations that meet both criteria. In contrast, isolation technologies based on a single principle, such as size-exclusion chromatography, cannot achieve the same level of purity as dual-principle approaches.

Moreover, ExoTFF’s combination of TFF and ExoFilter allows for the rapid processing of large sample volumes, providing high-throughput capacity for efficient, large-scale EV isolation from various biofluids.^16^ The system’s automated operation and fraction collection ensure consistent workflows and reproducible results across different sample types and volumes, making ExoTFF a robust solution that surpasses conventional EV extraction techniques.

While the ExoTFF system demonstrated substantial improvements in EV yield and purity compared to conventional methods, several considerations remain regarding the interpretation of these results. A major issue lies in the discrepancies among commonly used EV quantification methods, including nanoparticle tracking analysis (NTA), bicinchoninic acid (BCA) assay, immunocapture-based ELISA, and RT-qPCR. Each method evaluates different aspects of the EV population, particle number, protein content, surface marker presence, or internal nucleic acid cargo, leading to potential inconsistencies.

For example, label-free NTA cannot differentiate EVs from similarly sized particles such as LDL and VLDL, often resulting in overestimated particle counts. Likewise, BCA quantifies total protein without distinguishing between EV-associated and contaminant proteins, limiting its specificity. ELISA is subject to limitations in target accessibility and signal saturation, while RT-qPCR, although more selective, is not yet standard in routine EV workflows. These limitations highlight the urgent need for standardized, orthogonal quantification protocols.

A second critical challenge is the coisolation of lipoproteins, especially LDL and VLDL, which closely resemble EVs in both size (18–80 nm) and surface charge (−10 to −20 mV), and are present in much higher abundance in plasma. Because current isolation strategies rely predominantly on physical properties such as size or charge, they are inherently limited in selectively excluding such contaminants. This poses significant complications in downstream applications, particularly when lipoprotein-associated biomolecules interfere with proteomic or transcriptomic profiling. Future advances in EV isolation will likely depend on the development of affinity-based strategies that target lipoprotein-specific markers to achieve higher specificity. Incorporating such selectivity into scalable systems like ExoTFF would substantially enhance purity and reproducibility in EV analysis.

In light of these challenges, the recent emergence of fluorescence-enabled NTA and nanoflow cytometry (nano-FCM) represents a promising direction for more accurate and reliable EV quantification. These advanced tools can enable better discrimination of EVs from nonvesicular particles and facilitate more standardized evaluation of isolation techniques in terms of both yield and purity.

## Conclusions

In this study, ExoTFF demonstrated a 933% improvement in EV purity over conventional TFF, highlighting the synergistic effect of combining charge-based and size-based filtration. It also outperformed ultracentrifugation and TFF in isolating high-purity EVs from complex biofluids such as CCM, urine, and saliva. Given its high yield and compatibility with scale-up, ExoTFF presents a promising solution for industrial-scale EV production and diverse downstream applications in diagnostics and therapeutics.

## Supplementary Material



## Notes

Additional experimental details, materials, and methods, including optimization of TFF parameters, particle recovery data, and characterization results of EVs using NTA, TEM, Western blot, and RNA analysis (PDF)
