# Supplementary material for: Advanced Extracellular Vesicle Isolation: A Hybrid Electrokinetic-Tangential Flow Filtration Approach for Improved Yield, Purity, and Scalability
Source: Anal Chem. 2025 Jul 30;97(31):16759–68. doi: 10.1021/acs.analchem.5c01168 (PMC12355454; doi:10.1021/acs.analchem.5c01168)
Supplement: Supplementary file 1 [file ac5c01168_si_001.pdf]

Supporting Information

Advanced Extracellular Vesicle Isolation: A Hybrid Electrokinetic-Tangential Flow Filtration Approach for Improved Yield, Purity, and Scalability

YongWoo Kim[1‡], SoYoung Jeon[2‡], KangMin Lee[1], Sehyun Shin [1,2,3*]

[1] School of Mechanical Engineering, Korea University, Seoul, 02841, Republic of Korea

[2] Department of Micro-Nanosystem Technology, Korea University, Seoul, 02841, Republic of Korea

[3] Engineering Research Center for Biofluid Biopsy, Seoul, 02841, Republic of Korea

* To whom correspondence should be addressed:

Sehyun Shin

E-mail: lexerdshin@korea.ac.kr

‡These authors contributed equally.

Table of Content

Figure S1 Schematic of dual principles in ExoTFF 3

Figure S2 Protocol and operating principle of the syringe-based filtration system....... 4

Detailed Materials and Methods 5

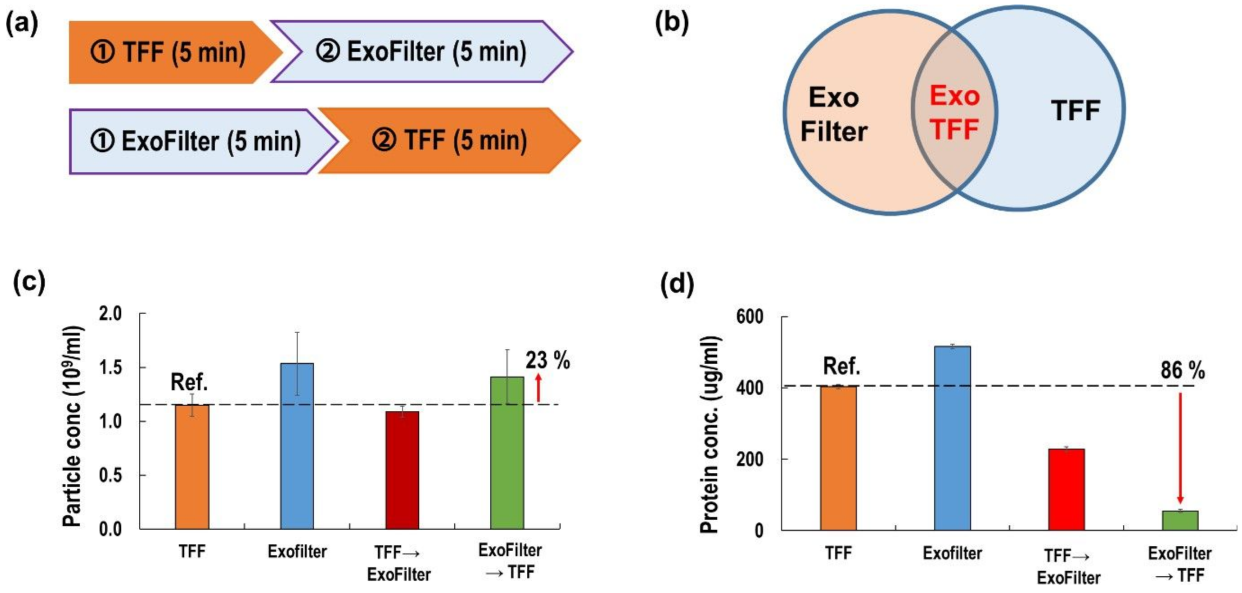

Figure S1. Schematic of dual principles in ExoTFF (a) Schematic representations of the order-dependent tests, comparing the sequence of TFF followed by ExoFilter and ExoFilter followed by TFF, (b) Application of dual principles in ExoTFF, (c-d) Results of the order-dependent tests, showing the effects on particle concentration and protein concentration from blood plasma.

In this study, while attempting to simply combine the two aforementioned technologies, we explored two different order-dependent tests: Comparison of TFF Followed by ExoFilter and ExoFilter Followed by TFF, as shown in Figures S1(a). This combined isolation method is expected to yield exceptionally pure EVs by applying two principles. The order of these techniques had little effect on the yield of EV isolation, but it had a significant impact on impurity removal. For instance, in the case of blood plasma (10 mL), treating the sample first with the ExoFilter and then with TFF led to a substantial reduction in protein concentration, achieving an 86% decrease compared to isolating EVs via TFF alone, as shown in Fig. S1(d). This result warrants more careful interpretation, as the underlying mechanism may involve multiple factors.

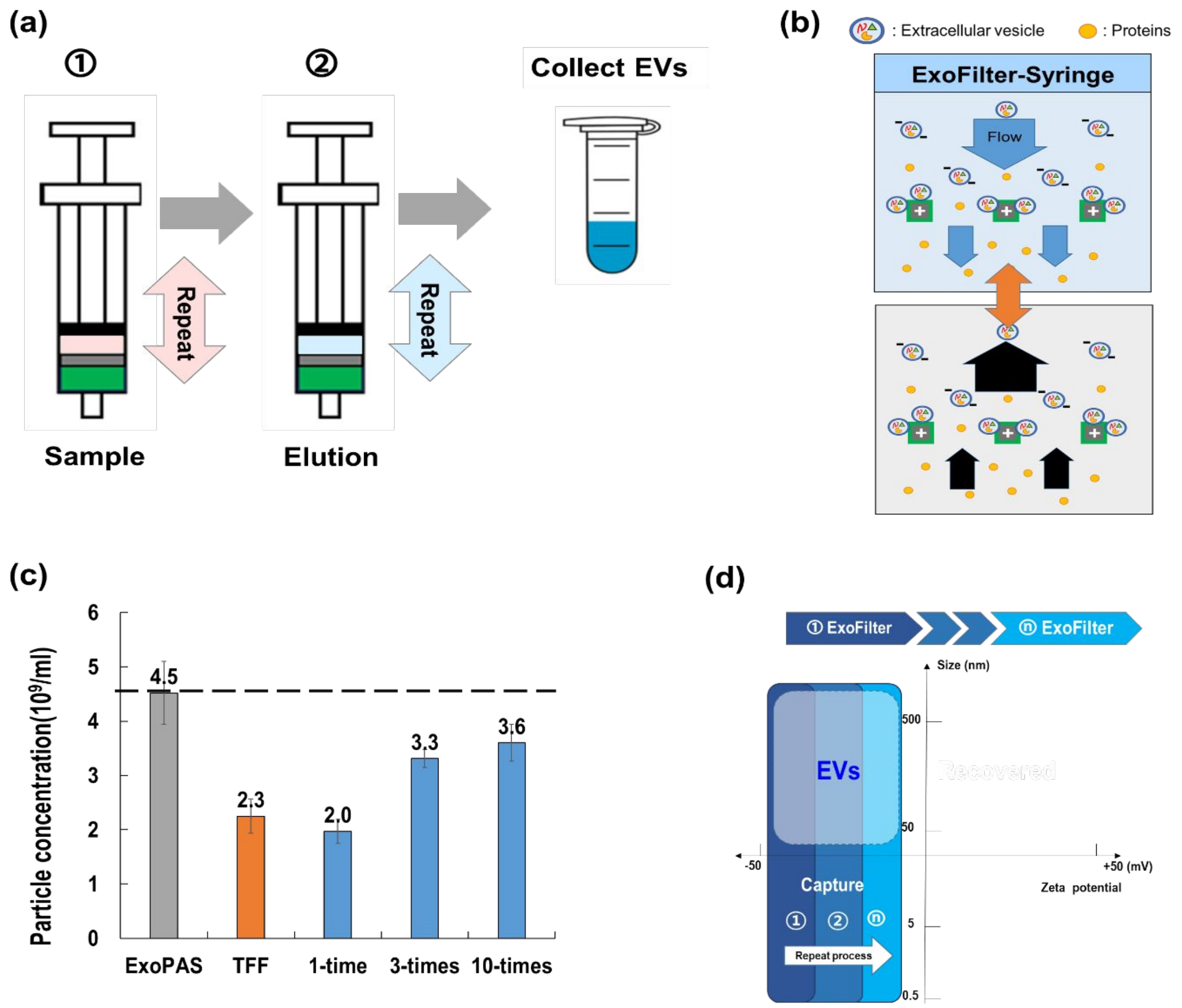

Figure S2. Protocol and operating principle of the syringe-based filtration system. (a) Workflow of the syringe-based filtration process, (b) Mechanism of particle capture in the syringe-based system, (c) NTA measurement showing increased particle concentration with repeated filtration cycles, (d) Effective EV isolation area within the filtration system.

Detailed Materials and Methods

Preparation of cationic polymer coated mesh Nylon meshes (Lixin Huarun MESH Co., China) were cut to 11 mm diameter using a laser cutter (BEAMO, MIRTECH Korea) and treated with protamine sulfate (Sigma-Aldrich, USA). First, the meshes were incubated in 0.1N hydrochloric acid for 30 minutes and washed with deionized water. They were then treated with 2.5% glutaraldehyde for 30 minutes, followed by additional rinsing. For activation, 5 mL of 0.1M EDC and NHS solutions were added, and the meshes were incubated for 1 hour before washing again. Finally, 100 mg of protamine sulfate dissolved in 10 mL of water was applied to the meshes, which were mixed for 15 minutes and left at room temperature for 6 hours. The coated meshes were rinsed three times to remove residual protamine sulfate.

Design of the Syringe-Type ExoFilter We developed a syringe-based ExoFilt er system, consisting of multi-layered meshes conjugated with cationic substanc es, securely anchored within a 10 mL syringe using a frit. The capacity of the syringe can be adjusted to accommodate different sample volumes, enabling th e production of various sizes of syringe-type ExoFilter. For this study, a 10 mL syringe was selected based on its compatibility with the minimum volume (5 mL) that can be processed by Tangential Flow Filtration (TFF), facilitating integ ration with the TFF system.

Preparation of various samples This study adhered to the ethical standards of the Declaration of Helsinki. Plasma samples were sourced from Zen-Bio Inc. (Research Triangle, NC, USA). Additional samples, including cell culture media from umbilical cord mesenchymal stem cells, urine, and saliva, were collected in sterile containers and purified through centrifugation. All samples were centrifuged at 3000 × g for 15 minutes to sediment and remove large particulates. The clarified supernatant was then passed through an 800 nm pore-sized mesh to exclude particles larger than 800 nm. Samples were subsequently stored at -80°C pending further analysis.

EV Isolation via ExoFilter The charge-based ExoFilter system was used to isolate EVs from plasma, urine, saliva, and CCM, with filters available in various capacities (1 mL, 3 mL, 15 mL, 200 mL) to suit different sample volumes. For smaller samples (1 mL, 3 mL, 15 mL), filtration was performed at 1 atm, followed by centrifugation at 5,000 × g for 1 minute, capturing negatively charged EVs through electrostatic interaction with

the positively charged mesh. Elution was achieved with 1 M NaCl, using 1/5 of the sample volume. For larger 200 mL samples, the flow rate was controlled with a vacuum pump at -68 kPa, followed by the same elution process. Protocol and operating principle of the syringe-based filtration system were depicted in Figure S2. The particle concentration increased progressively with each repeated process.

EV Isolation via Tangential Flow Filtration (TFF) For size-based filtration via tangential flow filtration (TFF), we utilized TFF-EVs (Hansa BioMed, Estonia) with an 800 kDa molecular weight cut-off and a fiber pore size of 50 ± 10 nm. We l oaded 10 mL of various biofluids into the TFF system for processing. The sam ples were circulated through the TFF device using a syringe, repeatedly passin g them back and forth until all the liquid was filtered. Following the initial filtrati on, a washing step involved circulating 10 mL of PBS in the same manner to cleanse the system. Finally, EV elution was performed by passing back and for th 2 mL of PBS through the TFF device back and forth to ensure comprehensi ve elution of the EVs.

EV Isolation via ExoTFF The ExoTFF system was used to isolate EVs from various samples, including plasma, urine, saliva, and cell culture media (CCM). During filtration, a 10 mL sample is loaded into the syringe-type ExoFilter and connected to the TFF system, with repetitive piston movements capturing negat ively charged EVs in the positively charged mesh. Impurities like lipoproteins a nd proteins smaller than 30 nm are removed via TFF. For elution, 10 mL of 1 M NaCl releases the captured EVs, with the process repeated until all liquid is expelled. In the recovery step, a new syringe with 2 mL of PBS recovers the EVs, concentrating them approximately fivefold.

EV isolation via Ultracentrifugation (UC) Ultracentrifugation (UC), the gold standard for EV isolation, is time-intensive, taking over 6 hours. For this process, 1 mL of biofluids was diluted 1:3 in PBS. The samples were centrifuged at 3,000 × g for 15 minutes, followed by 12,000 × g for 30 minutes to remove larger vesicles and debris. The supernatant was then ultracentrifuged at 120,000 × g at 4°C for 2 hours. After discarding the supernatant, the pellet was washed in PBS and spun again at 120,000 × g for 1 hour. The final pellet was resuspended in 200 μL of PBS for further analysis.

EV isolation via ExoQuick For polyethylene glycol (PEG)-based extracellular vesicle

(EV) isolation, the ExoQuick exosome precipitation solution (EXOQ5A-1; System Biosciences, Palo Alto, CA, USA) was used. A total of 252 μL of the ExoQuick solution was added to 1 mL of biofluid sample and incubated for 30 minutes at 4°C. After incubation, the sample was centrifuged at 1500 × g for 30 minutes, and the supernatant was carefully decanted, leaving the pellet in the tube. To ensure complete removal of the ExoQuick solution, an additional centrifugation at 1500 × g for 5 minutes was conducted. The pellet was then resuspended in 200 μL of PBS.

EV isolation via SEC EVs were isolated using a 70 nm qEV10 size-exclusion chromatography column (IZON Science, Cambridge, MA) and an automatic fraction collector-V2 (IZON Science, Cambridge, MA). Initially, the column was equilibrated at room temperature for 30 minutes and subsequently washed with phosphate-buffered saline (PBS). A 10 mL biofluid sample was then loaded onto the column. Seven consecutive 2 mL fractions were eluted by the addition of PBS. In accordance with the manufacturer's protocol, the fourth fraction, which was enriched in EVs, was utilized for further analysis.

Scanning Electron Microscopy (SEM) Imaging SEM imaging was performed using an anodic aluminum oxide (AAO) membrane to analyze EVs isolated via ExoTFF from plasma. +EVs were filtered through the membrane, fixed with glut araldehyde for 30 minutes, and dehydrated stepwise with ethanol (25–100%). A fter oven-drying at 37°C for 2 hours, the membrane was platinum-coated to en hance contrast. EVs and aggregates were examined using a Quanta 250 FEG SEM (FEI, USA).

Transmission Electron Microscopy (TEM) Imaging The Carbon Formvar Fil m-150 copper grid was handled with tweezers to ensure the sample side faced up and placed on a Petri dish. EVs isolated by ExoTFF from plasma were ap plied (15 μL) and left to adsorb for one minute, covered to prevent contaminati on. For negative staining, 1% uranyl acetate was applied vertically, and excess stain was blotted off. After air drying on filter paper, the grid was imaged using a JEM-1400 Flash TEM (JEOL Ltd., Japan) at 120 kV.

Nanoparticle Tracking Analysis (NTA) Nanoparticle Tracking Analysis (NTA) was performed using the NS300 system with NTA 3.4 Software (NanoSight, U K) to assess EVs. Samples were diluted in pre-filtered PBS, and three 30-seco nd

videos were recorded per sample with the camera level at 14 and detection threshold at 11. This analysis determined the EVs' average size and concentr ation based on the applied dilution factors.

Bicinchoninic Acid assay (BCA) Protein concentrations were measured using the Pierce™ BCA Protein Assay Kit (Thermo Scientific, USA) with a calibratio n curve prepared from bovine serum albumin (0–2000 μg/mL). Each standard a nd sample was measured in triplicate. A 100 μL sample was mixed with 2.0 m L of assay reagent, incubated at 37°C for 30 minutes, and allowed to cool to r oom temperature before absorbance was measured at 562 nm using a DS-11 spectrometer (Denovix, USA). Protein concentrations were calculated by compar ing sample absorbance to the standard curve.

Western Blotting Analysis Proteins for Western blot analysis were extracted from EVs suspended in 200 μL of elution buffer, mixed with Laemmli buffer an d 2-mercaptoethanol, and heated at 95°C for 10 minutes. SDS-PAGE was perf ormed using a Mini-PROTEAN® TGX™ Precast Gel (Bio-Rad, USA). Western blotting employed antibodies against EV markers CD9, CD81, TSG101, ALIX, A poA1, ApoB100 and albumin as a negative control. Antibodies included anti-CD 9, anti-CD81, anti-TSG101, anti-ALIX, anti-ApoA1, anti-ApoB100, and anti-albumi n (Abcam, UK) with goat anti-rabbit IgG as the secondary antibody. Protein ba nds were visualized using the ChemiDoc™ XRS+ System with enhanced chemi luminescence (ECL) detection.

Immunocapture-based ELISA Anti-CD9 (BioLegend, San Diego, CA), anti-CD81 (BioLegend, San Diego, CA), anti-ApoB100 (R&D Systems, Minneapolis, USA) and anti-ApoA1 (R&D Systems, Minneapolis, USA) antibodies were diluted to a concentration of 5 μg/mL in 10 mM PBS. 100μL of each diluted solution was added to microwells and incubated at 37°C for 2 hours. The wells were then washed with distilled water, followed by the addition of 200 μL of 0.5% casein in PBS, and incubated at 37°C for 1 hour. After additional washing, the 100μL samples were added to the wells, and the reaction was conducted 37°C for 2 hours. Following another rinse with distilled water, 100 μL of biotinylated anti-CD63 antibody (BioLegend, San Diego, CA),), biotinylated anti-ApoB100 (R&D Systems, Minneapolis, USA) and biotinylated anti-ApoA1 (R&D Systems, Minneapolis, USA)

diluted to 1 μg/mL was added to each microwell for a 1hour reaction. After a subsequent wash, 100 μL of 0.45 μm membrane-filtered streptavidin-poly HRP20 (Fitzgerald, Acton, MA, USA) at 66 ng/mL was dispensed into each well and incubated at 37°C for 1 hour. To develop the signal, 200 μL of HRP substrate solution was added to the wells and incubated at room temperature for 15 minutes. The reaction was terminated by adding 50 μL of 2 M sulfuric acid to each well. The absorbance was measured at 450 nm using a microplate reader (SPECTROstar Nano, BMG LABTECH, Freiburg, Germany).

miRNA Analysis via RT-qPCR To quantify specific miRNA markers within EV s, RNA extracted from the EVs was reverse transcribed using the TaqMan Mic roRNA RT kit (4366596, Life Technologies, Eugene, OR, USA) and analyzed w ith TaqMan MicroRNA Assays (4427975, Life Technologies, USA). The assay s pecifically targeted hsa-let-7a-5p and hsa-miR-142-3p, using TaqMan Universal Master Mix II without UNG (4440040, Life Technologies, Eugene, OR, USA).

Cellular Uptake Analysis Cellular uptake of EVs by human dermal fibroblasts (HDF) was assessed using EVs labeled with PKH67 green fluorescent dye (Sig ma-Aldrich, Burlington, MA, USA). The labeling was conducted for 15 minutes at 25°C, followed by the removal of excess dye through a 100-kDa filter. The HDF cells were then exposed to PKH67-stained EVs at a concentration of 2 × 10^9 particles/mL in their culture medium. Nuclear staining was achieved by addi ng Hoechst 33342 dye to the medium, and the cellular uptake of EVs was vis ualized using a fluorescence microscope (Eclipse Ti2; Nikon, Tokyo, Japan).

Cytotoxicity test To evaluate cell viability, HDF cells were seeded in 96-well plates at a density of 1 × 10^4 cells/cm² and allowed to adhere for 24 hours. Following this, the cells were washed and then treated with EV-depleted FBS-supplemented medium along with EVs at a concentration of 2 × 10^9 particles/mL for 72 hours. Cell viability was evaluated using a WST-1 assay kit (EZ-Cytox; DoGenBio, Seoul, Korea). The WST-1 reagent was mixed with the culture medium in a ratio of 1:10, and 100 μL of the mixed solution was added to each well of the 96-well plate. The cells were incubated at 37°C for 1 hour before the absorbance at 450 nm was measured to ascertain viability.

Zeta potential analyzer Zeta potentials for EVs and various plasma proteins including albumin, γ-globulin, and fibrinogen were determined using a Zetasizer Pro (Malvern

Panalytical, Malvern, UK). Given the challenges associated with resuspending EVs and plasma proteins in deionized water, each 10-μL sample was diluted in 990 μL of deionized water prior to measurement. The cationic nylon mesh was analyzed using a Surpass 3 analyzer (Anton Paar GmbH, Austria), employing a protamine-conjugated mesh measuring 20 mm by 10 mm for this purpose.
